# Integrative analysis of genetic and epigenetic profiling of lung squamous cell carcinoma (LSCC) patients to identify smoking level relevant biomarkers

**DOI:** 10.1186/s13040-019-0207-y

**Published:** 2019-10-21

**Authors:** Bidong Ma, Zhiyou Huang, Qian Wang, Jizhou Zhang, Bin Zhou, Jiaohong Wu

**Affiliations:** 1 Department of Medical Oncology, Zhe Jiang Chinese Medicine University affiliated Chinese Medicine Hospital, Wen Zhou, Zhe Jiang province People’s Republic of China; 2 Tianjia Genomes Tech CO., LTD., No. 6 Longquan Road, Anhui Chaohu economic develop zone, Hefei, 238014 People’s Republic of China; 3 Department of Gynecology and Oncology, Wen Zhou Medical University affiliated People’s Hospital, Wen Zhou, Zhe Jiang province People’s Republic of China

**Keywords:** Lung squamous cell carcinoma, Data mining, RNA-seq, Methylation, The Cancer genome atlas, Smoking intensity

## Abstract

**Background:**

Incidence and mortality of lung cancer have dramatically decreased during the last decades, yet still approximately 160,000 deaths per year occurred in United States. Smoking intensity, duration, starting age, as well as environmental cofactors including air-pollution, showed strong association with major types of lung cancer. Lung squamous cell carcinoma is a subtype of non-small cell lung cancer, which represents 25% of the cases. Thus, exploring the molecular pathogenic mechanisms of lung squamous cell carcinoma plays crucial roles in lung cancer clinical diagnosis and therapy.

**Results:**

In this study, we performed integrative analyses on 299 comparative datasets of RNA-seq and methylation data, collected from 513 lung squamous cell carcinoma cases in The Cancer Genome Atlas. The data were divided into high and low smoking groups based on smoking intensity (Numbers of packs per year). We identified 1002 significantly up-regulated genes and 534 significantly down-regulated genes, and explored their cellular functions and signaling pathways by bioconductor packages GOseq and KEGG. Global methylation status was analyzed and visualized in circular plot by CIRCOS. RNA-and methylation data were correlatively analyzed, and 24 unique genes were identified, for further investigation of regional CpG sites’ interactive patterns by bioconductor package coMET. AIRE, PENK, and SLC6A3 were the top 3 genes in the high and low smoking groups with significant differences.

**Conclusions:**

Gene functions and DNA methylation patterns of these 24 genes are important and useful in disclosing the differences of gene expression and methylation profiling caused by different smoking levels.

## Background

Lung cancer ranked the second place in estimated new cases (approximately 234,000 new cases) and the first place in estimated deaths (approximately 160,000 new cases) in United States, 2018 [1]. Recent studies reveal several major risk factors influence the lung cancer [2, 3]. For example, environmental pollutions and tobacco consumption [4–6]. Chronic exposure to carcinogens released by smoking metabolically induced damages on pulmonary cells and caused DNA mutation or DNA adducts, eventually leading to lung cancer [7, 8]. Seeking the connection between smoking habits and changes of genetic and epigenetic levels in all types of lung cancer is quiet promising in finding unique biomarkers for diagnosis and treatment, especially after people found out smoking showed various impacts of different types of lung cancers [9].

There are two main types of lung cancers, small-cell lung carcinoma (SCLC) and non-small-cell lung carcinoma (NSCLC). Lung squamous cell cancer (LSCC) is the second common subtype of the latter, which represented almost 25% of overall lung cancer [10]. Numerous studies had focused on genomic characteristics of NSCLC (including LSCC), especially mutated genes found in lung cancer patients. Glutathione S-Transferase Mu 1 (GSTM1) is a glutathione S-transferase, which functions as detoxifier in tobacco smoking induced carcinogenesis [11]. GSTM1 deficiency would significantly increase the risk of LSCC [12]. Cytochrome P450 Family 1 Subfamily A Member 1 (CYP1A1) was also found high polymorphic and associated with elevated risk of lung cancer [13, 14]. Epidemiology studies showed that there was an increasing risk of developing lung cancer in cigarette amount dependent manner on both GSTM1 and CYP1A1 gene mutate patients [15]. Other genes with gain or loss of function mutations, such as FGFR1, TP53 and NOTCH1, might also play important roles in developing LSCC [16–19]. The TCGA project conducted a more comprehensive study on the genomes and regulatory pathways of LSCC, and reported more mutated genes, such as CDKN2A, PTEN, PIK3CA, NFE2L2, KEAP1 and RB1 [20]. However, compared to lung adenocarcinoma, the understanding of mutated genes and pathways in LSCC is still limited, especially in terms of actionable mutations.. Next-generation sequencing and gene chip technology produce high-throughput RNA, DNA and CpG methylation data from multiple LSCC patients. With appropriated bioinformatics analysis tools, these data would be revisited/reinvestigated in a more comprehensive perspective of gene differentiation and regulatory pathways, and for the purpose to identify significant biomarkers for diagnosis and treatment.

In this study, we aimed to identify biomarker genes related to the smoking intensity in LSCC patients, and performed the integrative analysis using dataset downloaded from The Cancer Genome Atlas (TCGA). In particular, gene ontology, cellular enrichment functions and pathway analysis were performed on RNA-seq data. Global DNA methylation profiles were drawn from on methylation data. Significant biomarker genes were selected and regional CpG methylations of them were evaluated. All the bioinformatics analyses were performed using Bioconductor packages and computer software. This study is for the first time to systematically explore the genetic and epigenetic profiles of LSCC patients and look for potential genes significantly different between high- and low-smoking groups. Such information is highly valuable for establishing an effective non-invasive screening method and treatment against human LSCC.

## Materials and methods

### Data source, RNA-seq and DNA methylation analysis

We used publicly available data for the TCGA-LSCC cohort that includes 513 cases [21, 22]. From this cohort, we obtained 299 LSCC cases with both gene expression and epigenetic datasets. We divided these data into two equal groups according to smoking intensity, 150 high smoking cases (average number of cigarettes consumed per year 73.6 packs with a median of 63 packs) and 149 low smoking cases (average number of cigarettes consumed per year 30.9 packs with a median of 30 packs).

The gene expression data was obtained as raw count values from TCGA public level 3 transcription profiles. We applied bioconductor package (edgeR) [23] to the transcription profiles and tested for differential expression between high-smoking and low-smoking groups. *P*-values were corrected for multiple testing by computing q-values (false discovery rates). Then the significant Differentially Expressed Genes (DEGs, *P* < 0.01 and fold change value > 2 or < − 2) were selected for further analysis. Principle component analysis was performed to classify between high- and low-smoking groups. The Gene Ontology (GO) functional annotation of DEGs was accomplished by Biomart Database and Kyoto Encyclopedia of Genes and Genomes (KEGG) pathway annotation of DEGs was accomplished by using BLASTP to align to KEGG database with a cutoff e-value of 10–5 [24–26]. GO enrichment analysis provides all GO terms that significantly enriched in DEGs comparing to the genome background, and filtered the DEGs that correspond to biological functions. This method firstly mapped all DEGs to GO terms in the database [27], calculating gene numbers for every term, then using hyper geometric test to find significantly enriched GO terms in DEGs comparing to the genome background. The formula is:

$$ P=1-\sum \limits_{i=0}^{m-1}\frac{\left({}_i^M\right)\left({}_{n-i}^{N-M}\right)}{\left({}_n^N\right)} $$

Where N is the number of all genes with GO annotation; n is the number of DEGs in N; M is the number of all genes that are annotated to the certain GO terms; m is the number of DEGs in M. The calculated *P*-value goes through Bonferroni Correction, taking corrected P-value < = 0.05 as a threshold. GO terms fulfilling this condition are defined as significantly enriched GO terms in DEGs. Pathway enrichment analysis identified significantly enriched metabolic pathways or signal transduction pathways in DEGs comparing with the whole genome back ground. And the calculating formula was the same as that in GO enrichment analysis.

The DNA methylation data was obtained as beta values from TCGA public level 3 methylation profiles. We applied bioconductor packages *CHAMP* to the methylation profiles and tested for differential methylated CpG site and region between smoking high and low group samples [28]. Individual samples and CpG sites with a high missing rate (> 5%) were excluded. The overall DNA methylation status was analyzed and visualized by CIRCOS, a multi-layer highly informative circular infographics suitable for different sets of DNA methylation status [29].

### Integrative analysis of RNA-seq and DNA methylation data

Integrative analysis of RNA-seq and DNA methylation data was performed and detected *cis*-related correlations of CpG methylation and RNA expression. The core set of samples was used since all samples in this set had data available across the two platforms. For analysis involving the RNA-seq datasets, a log_2_-transformation was used in order to deal with skewness in the data. Filtered DEGs from both datasets were picked up and showed in a Column/Dot plot with RNA expression and DNA methylation status. Given the scientific evidence that reverse regulation was shown between DNA methylation and transcription levels, all genes that following the relationship were selected for further detailed pattern analysis.

### Identification of regional DNA methylation pattern on selected genes

For identifying the regional DNA methylation pattern from selected 24 genes, bioconductor package *coMET* was used for analysis and data visualization [30]. The *coMET* package provides information including chromosome position of genes, regional CpG site methylation changes, genomic annotation tracks and correlation of DNA methylation in selected CpG sites. The *coMET* is valuable for interpreting paternal results in order to fully elucidate the relationship between gene epigenome and transcriptional expression.

## Results

### Differentially expressed genes between LSCC patients in high and low groups

In order to explore the genetic profiles and identify significant genes differentially expressed in the comparison of high and low groups, we performed principle component analysis (PCA), DEGs discovery and statistical analysis (EdgeR), Gene Ontology (GO) and KEGG analysis on all 299 RNA-seq datasets. First of all, PCA plot shows less significant variance between high and low groups and results are summarized in Additional file 1: Fig. S1. Even through the overall transcriptome signatures are not significantly different, it is very intriguing to see if individual genes are able to be identified to distinguish two groups by bioinformatical analysis. Using EdgeR, we identified a total number of 1002 up-regulated and 534 down-regulated DEGs in high smoking intensive group when compared to low group. Then we input up-regulated and down-regulated genes separately into *Goseq* package for GO and KEGG enrichment analysis to determine gene functions and pathways.

**Fig. 1:**
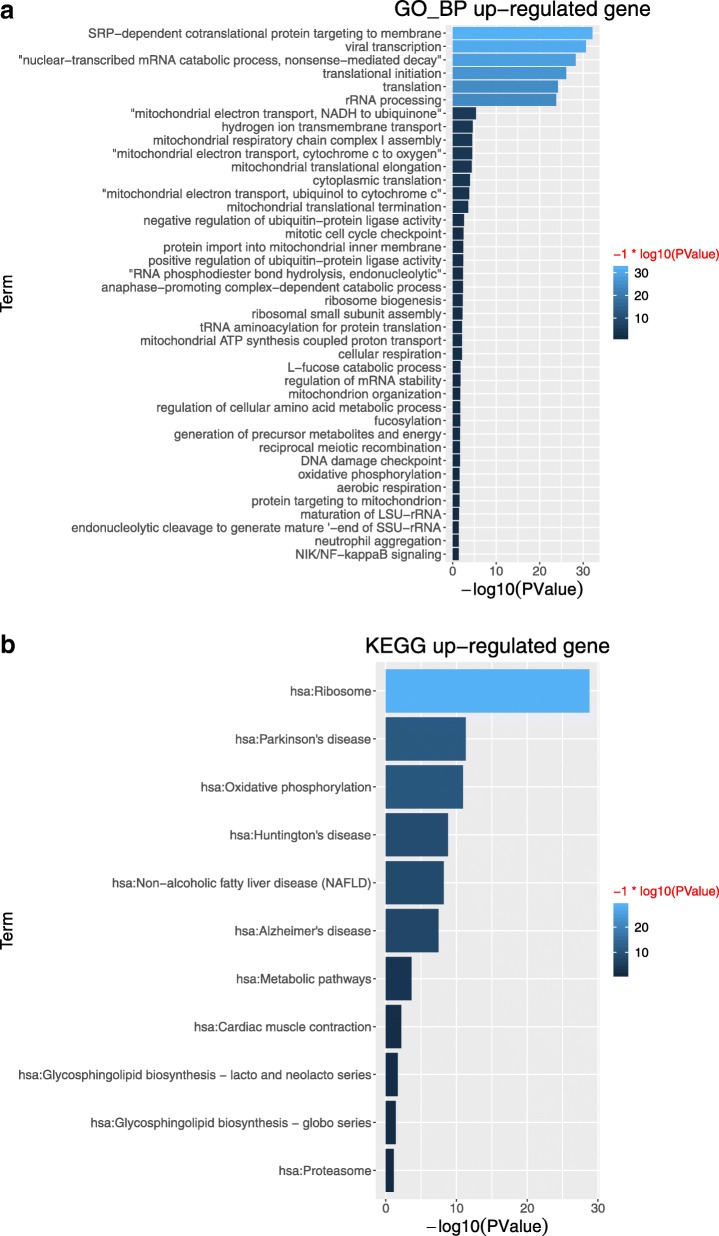
For up-regulated genes, GO terms and KEGG pathways. (**a**) Top ranked 40 enriched GO terms associated with biological process (**b**) 11 KEGG enrichment pathways. Log_10_ fold-changes are used as parameters

For up-regulated genes, top ranked 40 enriched GO terms and 11 KEGG pathways are summarized in Fig. 1a and b, respectively. In Fig. 1a, highly enriched GO terms with high gene numbers annotated can be roughly divided into two biological functions: regulating the mRNA formation and translation in ribosome which includes translational initiation, translation, rRNA processing, nuclear-transcribed mRNA catabolic process, nonsense-mediated decay, and viral infection to host cells (viral transcription). Several biological process GO terms such as mitochondrial electron transport, cytochrome c to oxygen, mitochondrial electron transport, NADH to ubiquinone are highly associated with mitochondrial membrane potential, which suggested that high intensive smoking might cause significant dysfunction of mitochondria. Mitochondrial membrane potential has been determined as a unique biomarker for oxidative environmental stress, especially for smoking cigarettes [31]. Intensive researches have been conducted and found out cigarette smoke not only induces significantly mitochondrial membrane potential alteration, but also disturbs the expression and distribution of membrane protein, mitochondrial depolarization [32, 33]. Reactive oxidative stress (or inflammation) associated biological process GO terms, NIK/NF-kappa B signaling, DNA damage checkpoint are also found. Nicotine has been reported to induce inflammation and oxidative stress through regulating the nuclear factor (NF)-kappa B signaling pathway [34]. DNA damage is also one of major carcinogenic mechanisms induced by smoking [35]. In Fig. 1b, 11 KEGG pathways are listed and ribosome pathway (and RNA transport pathway) are highly associated with biological process GO terms identified previously. Proteasome pathway involves in many cellular functions including regulating cell cycle [36], inflammation response [37] and induction of apoptosis [38] reported in cigarette smoking induced carcinogenesis. The summary of GO terms and KEGG pathways for up-regulated genes are summarized in Additional file 5: Table S1A and Additional file 6: Table S1B.

**Fig. 2:**
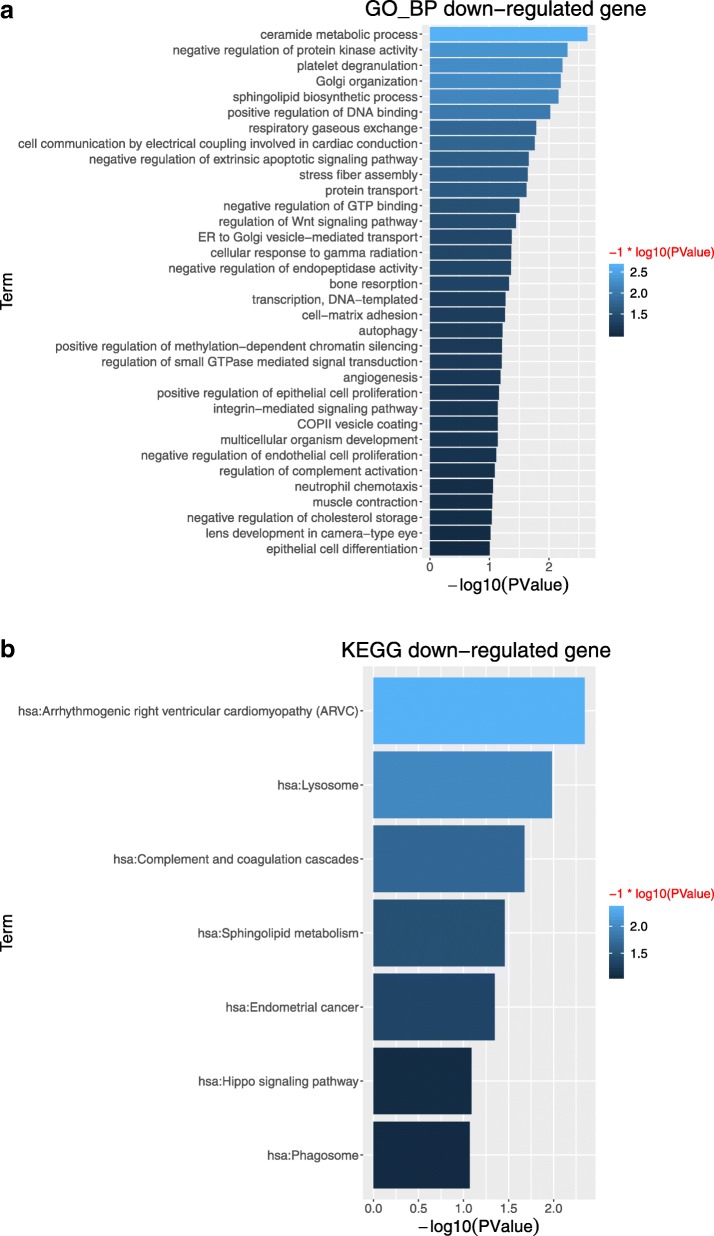
For down-regulated genes, GO terms and KEGG pathways. (**a**) Top ranked 34 enriched GO terms associated with biological process (**b**) 7 KEGG enrichment pathways.. Log_10_ fold-changes are used as parameters

For down-regulated genes, top ranked 34 enriched GO terms and 7 KEGG pathways are summarized in Fig. 2a and b, respectively. In Fig. 2a, transcription, DNA-templated is defined as regulating the RNA synthesis using DNA template. For the first time, high intensive smoking has been identified not only to positively affect RNA expression and function in ribosome, but also negatively influence the transcription using DNA-template. These findings are critical for fully understanding the mechanism of high cigarette smoking caused LSCC and other type of lung cancers, and provides guidance for future lung cancer research focusing on RNA function targeted therapies and diagnosis biomarkers. Regulation of Wnt signaling pathway, positive regulation of epithelial cell proliferation, negative regulation of apoptotic signaling pathway, negative regulation of endothelial cell proliferation can be grouped as cell fate signaling pathways. Wnt signaling pathway has been well studied as an important regulatory pathway response to cigarette smoking [39–42], and some of studies reports that Wnt pathway plays important roles in epithelial cells proliferation [43–45]. Smoking also impairs the endothelial cell proliferation through reactive oxidative stress, and inhibited cell apoptosis [46–48]. In Fig. 2b, phagosome is described as defendant system against infectious toxin induced tissue damage. Several studies show that inhalation of toxic particles during smoking is able to induce severe damages on cellular phagosome [49, 50]. Lysosome is main organelle responsible for external macromolecules degradation. Cigarette smoking is reported to impair the function of lysosome through dysfunction of NRF-2 antioxidant pathway [51] and immune-response apoptotic pathways [52], which pathways are well organized as critical regulators in many human cancer carcinogenesis [53, 54]. Hippo signaling pathway regulates the cell density and population by enhancing cell proliferation and inducing apoptosis, controlling organ size across many species including human [55]. Cohort studies suggest that hippo signaling pathways are involved and important in types of lung cancers [56]. Yes Associated Protein 1 (YAP1) and WW Domain Containing Transcription Regulator 1 (WWTR1/TAZ) are key regulatory factors of Hippo Signaling pathways, and are targeted to develop effective lung cancer therapy by their inhibition [57]. The summary of GO terms and KEGG pathways for down-regulated genes are summarized in Additional file 7: Table S2A and Additional file 8: Table S2B.

### DNA methylation status visualization

**Fig. 3:**
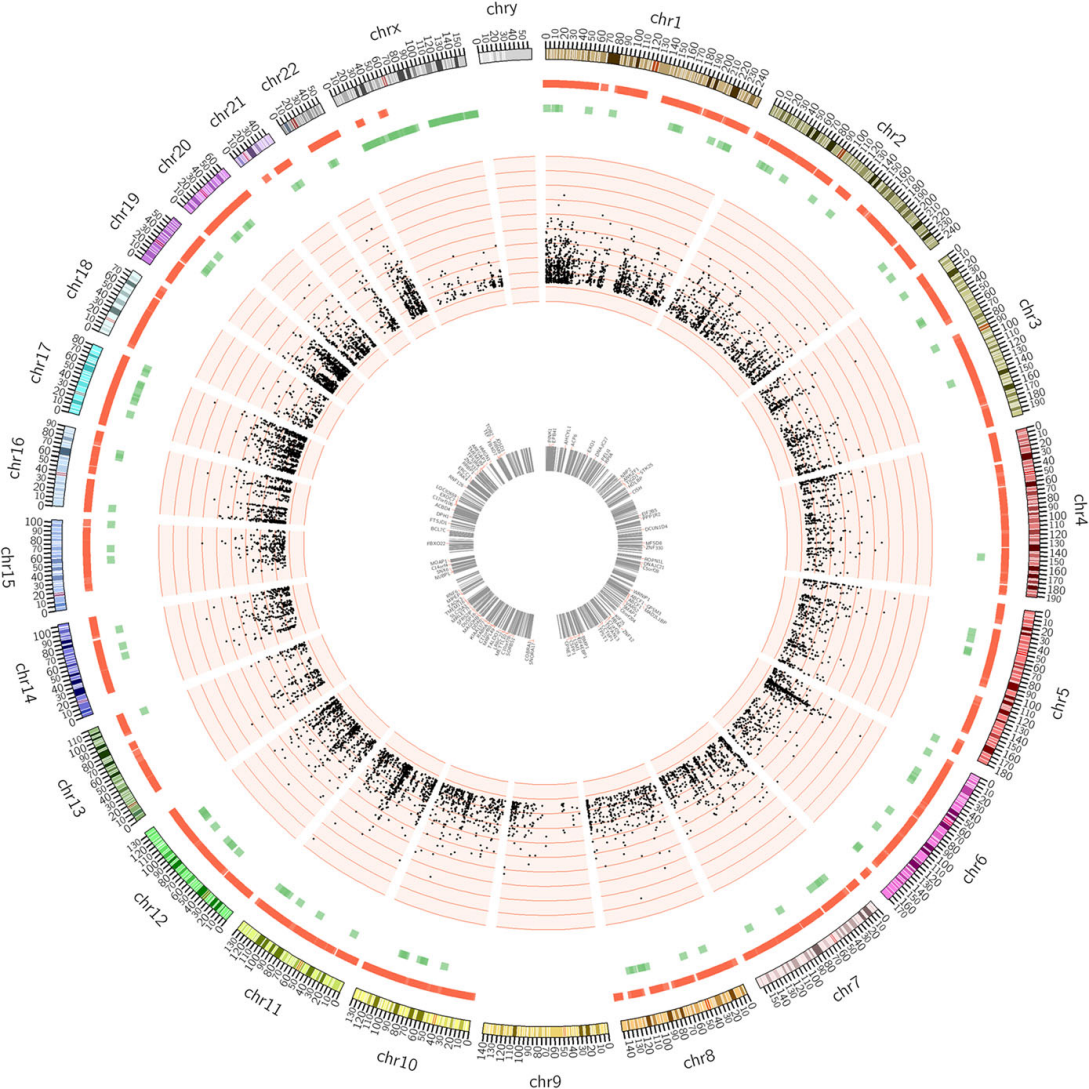
Circos analysis of global methylation status differences between high and low intensive smoking LSCC patients. The outer layer represents CpG islands heatmap. Red color represents CpG islands are hyper-methylated. Green color represents CpG islands are hypo-methylated. The middle layer represents scatter plot of single CpG site. Each dot represents a significant different DNA methylation changes, with *p*-value associated at Y-axis. The inner layer of circular plot is top 100 genes with the largest betafc value in DNA methylation changes of CpG islands

All 299 cases of methylation data were analyzed by Bioconductor package *CHAMP*. Beta value distribution and hierarchical cluster is summarized in Additional file 2: Figure S2A-B. The principle component analysis of methylation status difference between high and low groups is summarized in Additional file 3: Fig. S3. These Figs suggest that global epigenome of LSCC patients with different smoking intensity has few significant changes in this cohort study. This observation shows consistency with our previous results from RNA-seq dataset analysis. However, significant changes of individual differentially methylated CpG site and region between high and low group are identified by *CHAMP* package*.* To check overall genome-wide methylation changes, CIRCOS is used to visualize data as multiple-layer circular plot in Fig. 3. The outer layer contains information includes chromosome, CpG island position and DNA methylation alteration. The middle layer is scatter plot of CpG site location. Each marker represents a CpG site which can be either hypo- or hyper-methylated and Y-axis represents the possibility. The inner layer lists the top 100 gene which show the most significant CpG island methylation changes based on betafc values.

### Integrative analysis for biomarker gene identification

**Fig. 4:**
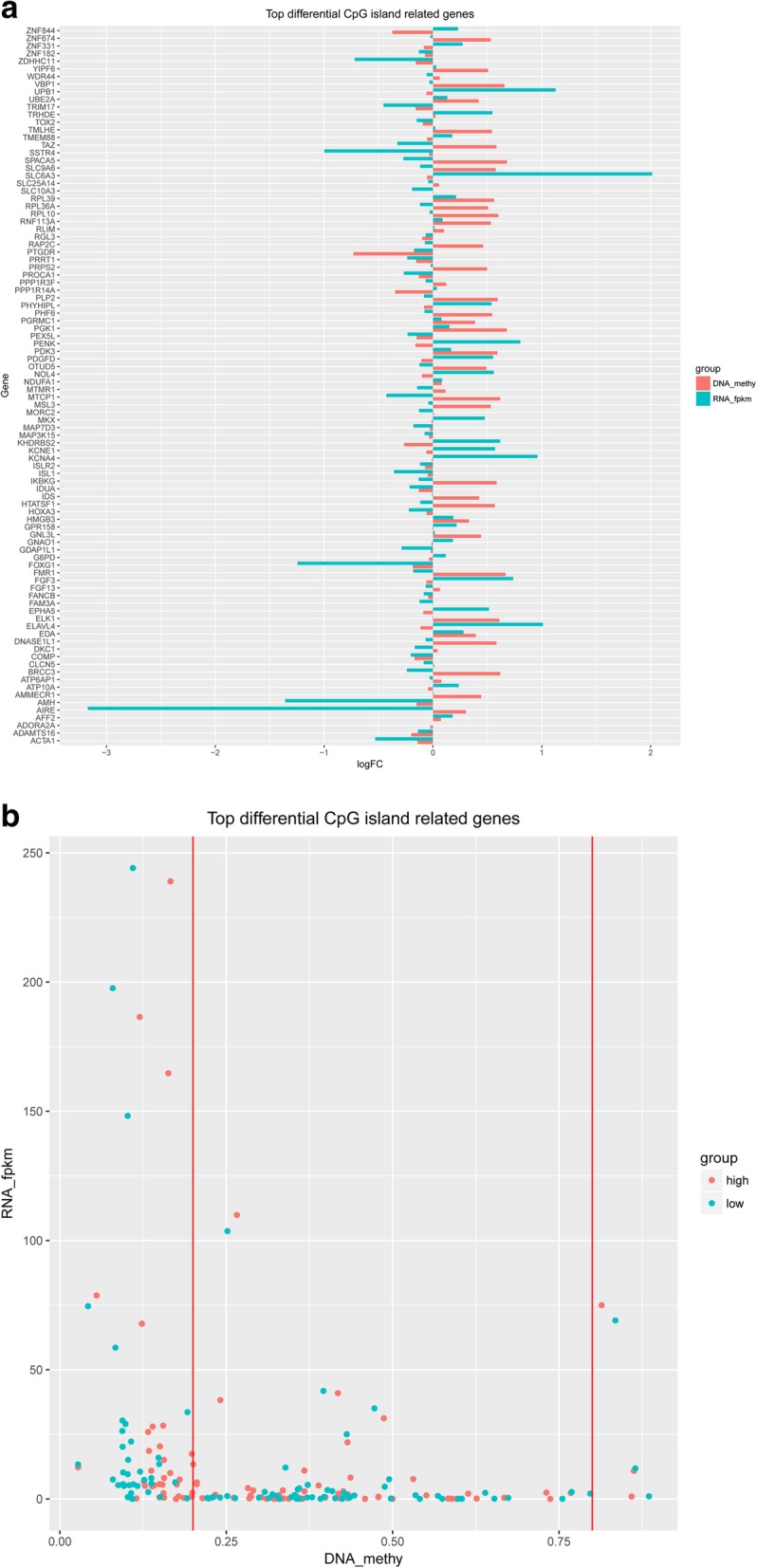
Top 50 significantly hyper/hypo methylated CpG islands annotated on 94 unique genes. (**a**) Represents DNA methylation status and gene ID in column plot (**b**) Represents DNA methylation status and gene ID in dot plot

The first step of integrative analysis is to select common genes showed in both RNA-seq and DNA methylation datasets, after gene filtration using core set presented previously in this study. First of all, top 50 hyper and hypo-methylated CpG islands on 94 genes were selected from methylation data, with a *p*-value < 0.05. They are summarized and visualized in Fig. 4a and b. In Fig. 4a, Column growing to right means CpG island (red column) hyper-methylated and gene expression (green column) increased. Column growing to left means CpG island hypo-methylated and gene expression decreased. Evidentially, genes with red and green columns growing to different directions are collected for further analysis. In Fig. 4b, all 94 genes expression and CpG island methylation status data from both high and low groups are extracted and presented as scatter plot. All dots close to right bottom corner are described as gene, which expression is lower and associated CpG island is hyper-methylated. These genes are considered having the most biological meaning in gene transcriptional pattern and potential to distinguish the difference between high and low groups.

**Fig. 5:**
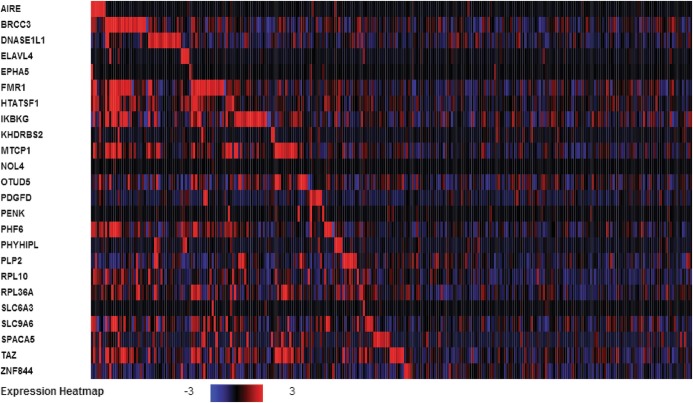
Heatmap of global transcriptional expression of 24 selected genes in LSCC patients. The expression (log2 fold-change) is scaled at the range of − 3 to 3. Blue represents gene expression being inhibited. Red color represents gene expression being induced

In order to further explore the genomic and epigenomic difference between high and low group, the second step of integrative analysis is to select gene with high transcriptional expression and low CpG island methylation status, or low transcriptional expression and high CpG island methylation status. 24 genes have been found to match such requirements and their transcriptional expression heatmap across all LSCC patients are presented in Fig. 5. In Fig. 5, Y-axis is 24 gene names and X-axis is LSCC patient RNA expression level arranged by clustering order. The expression scale is set up at log2 (fold-change) from − 3.0 to 3.0. From the heatmap, it is clear that some LSCC patients show positively correlated and other LSCC patients show negative correlation on the transcriptional level. This interesting finding leads us to further studying gene promoter region of these 24 genes CpG methylation correlation pattern in order to elucidate the possible reasons. In addition, the integrative analysis of DNA methylation status and transcriptional expression of 24 genes are also presented in Additional file 4: Figure S4A-B.

### DNA methylation correlation analysis on specific biomarker genes

**Fig. 6:**
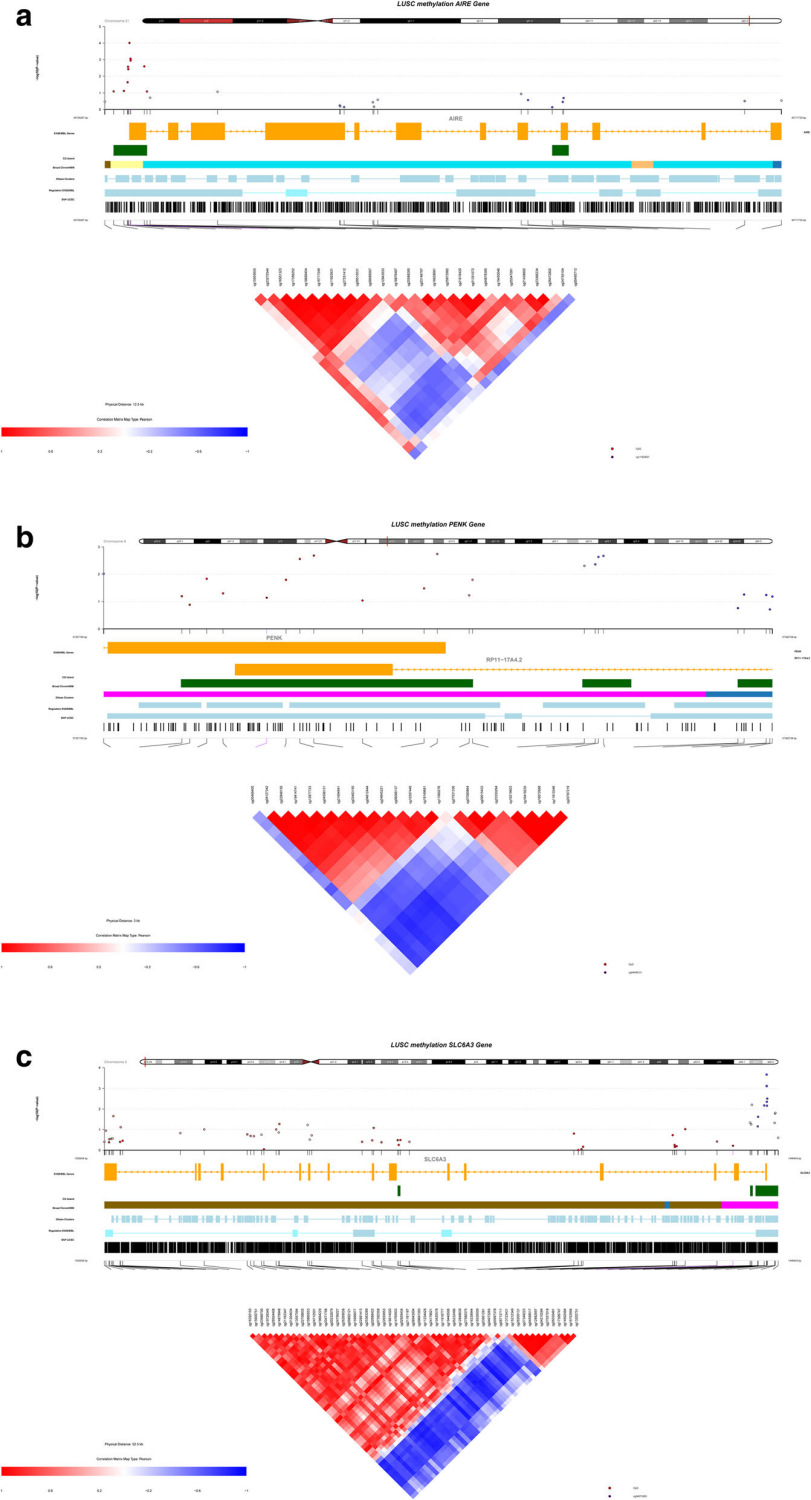
CoMET plot showing the regional CpG methylation patterns on gene AIRE, PENK and SLC6A3. (**a**) AIRE, (**b**) PENK, (**c**) SLC6A3 are organized by p-value, gene location, CpG island (green), Gene ENSEMBL (yellow), Broad ChromHMM (pink), DNase Clusters, SPN from UCSC etc. and the correlation heatmap of Spearman correlation values among CpGs in the gene regulatory regions

Methylation alternation in nearby CpG sites shows strong correlation and has been identified in recent studies [58–60]. CoMet package is used to graphically display regional CpG site methylation changes and related information. Autoimmune Regulator (AIRE), Proenkephalin (PENK) and Solute Carrier Family 6 Member 3 (SLC6A3) are top 3 genes in the integrative analysis above, and may show potential for clinical diagnosis and therapies of LCSS. Their information are summarized in Fig. 6a, b, and c respectively. The entire coMet Fig contains three layer: top layer shows chromosome position of gene, and CpG site methylation changes with –log (*p*-value) plot. Each dot represents a CpG site. Higher dot means this CpG site has more chance to be methylated. Middle layer contains information like gene ENSEMBL, CpG islands, Broad ChromHMM, SNP from USCS etc. The bottom layer shows the CpG site name and heatmap of nearby CpG site methylation correlation. The scale of heatmap is from − 1.0 to 1.0, in which − 1.0/blue means negatively correlated and 1.0/red means positively correlated. For AIRE gene, cg09510531 to cg00495713, and cg01351072 to cg18876487 show strong positive correlation. CpG site range cg11923631 to cg27251412 shows strong negative correlation with CpG site range cg04878385 to cg21616420. For PENK gene, cg04612444 to cg06066137, and cg11060276 to cg27531336 show strong positive correlation. CpG site range cg04612444 to cg00468400 shows strong negative correlation with CpG site range cg11060276 to cg27531336. For SLC6A3, cg04073265 to cg15999077 and cg17306747 to cg27580375 show strong positive correlation. CpG site range cg04073265 to cg16526509 shows strong negative correlation with CpG site range cg16614020 to cg27580375.

## Discussion

In this study, we found 24 identical genes in LSCC patients, which exhibit significant epigenetic and genetic patterns between high and low intensive smokers. AIRE, PENK and SLC6A3 are Top 3 candidate genes, may be involved in the development of LSCC. AIRE is a transcriptional regulator gene, which guides the production of tissue specific antigens [61, 62]. Mutation of AIRE genes caused the dysfunction of T cell development and the immune system self-tolerance breakdown [63]. Recently, AIRE genes are found expressed in both human androgen-sensitive prostate cancer LNCaP cells and human androgen-insensitive prostate cancer PC-3 cells [64]. Interleukin-6 (IL-6), which acts as both pro-inflammatory and anti-inflammatory cytokine, is proposed to be regulated directly by AIRE genes in PC-3 cells. In addition, AIRE is found highly expressed in PC-3 cells and functions as prostate tumor promoter gene in vivo. AIRE deficiency finds to decrease the expression of Tyrosinase Related Protein 1 (TYRP1) in thymus, yet increases the T cell immune responses against melanoma development [65]. AIRE is also involved in the development of tumor-associated Foxp3 + regulatory T cells (Tregs), which do not respond to tumor-specific antigens [66]. Epidemiological study shows that AIRE gene expression is strongly associated with tumor prognosis in ER positive breast cancer patients [67]. There is no direct evidence to demonstrate the role of AIRE in the development of human lung cancer, however based on your observations in this study and previous research, it is highly possible that AIRE regulates the transcriptional proteins overexpressed to induce G1 cell cycle arrest and apoptosis, and may also be indirectly involved in the immune escape of tumor cells. PENK (also known as proenkephalin A) regulates the expression of a serious of opioid polypeptides which modulates the immune system [68]. The mRNA level of PENK gene is significantly induced by the activation of T-Helper cell [69]. It has been identified that PENK was associated with has-miR126, which was found significantly down regulated in NSCLC [70]. The hyper-methylation of PENK was also found significantly in prostate cancer, colorectal cancer and lung adenocarcinoma [71–73].. Research suggests that PENK gene may be involved in activating the NF-kB and p53 pathways by transcriptional repression [74]. SLC6A3 is dopamine transporter encoding gene, which plays important roles in neuronal disorder diseases [75–78]. It was found that the polymorphisms of SLC6A3 was highly associated with body mass index [79], which the later one have been strongly suggested as risk factor of many types of cancer including lung [80–82]. Recently, SLC6A3 is proposed as poor prognostic biomarker in human triple negative breast cancer tissues [83]. Methylation status in promoter region of SLC6A3 gene shows significant strong correlation with overall survival, with significant probability difference over 40%. SLC6A3 is also found highly expressed in clear cell renal cell carcinoma, which indicates as powerful therapeutic biomarkers for human renal cancer diagnosis and treatment [84]. Moreover, SLC6A3 c.-1476 T > G polymorphism may increase the risk of NSCLC, and the gain of 5p15.33 (which containing SLC6A3) is the most frequent genetic event in early stage NSCLC [85, 86]. It suggests that SLC6A3 may play an important role in the development of LSCC.

Integrative analysis of complex transcriptome, genome, methylome, proteome dataset and clinical profiles for cancer research are widely applied [87–91]. Whole-exome sequence reveals several driver mutations, including TP53, PTEN, NFE2L2 and KEAP1, whether in White or Asian LSCC patients [92]. Another cohort study determines that BRF2 RNA Polymerase III Transcription Initiation Factor Subunit (BRF2*)* is a novel oncogene showing strong lineage association with specificity of LSCC in an integrative analysis of genomic and gene expression microarray profiles [93]. The WWTR1/TAZ gene is also identified by genomic, epigenomic and proteomic integrative analysis in LSCC cohort study [94]. Most researches are separating LSCC patients by smokers or not, but not significantly focusing on smoking habit (intensity) or regional CpG methylation pattern analysis. Therefore, for the first time, we categorized the LSCC patients by smoking intensity and divided them as high (> 59 packs per year) and low (< 38 packs per year) group in this study. We performed computational analysis to reveal RNA-seq and DNA methylation datasets with clinical records obtained from TCGA database. Transcriptome dataset are analyzed by multiple Bioconductor packages including EdgeR, Goseq and KEGG. Epigenetic datasets are analyzed by CHAMP Bioconductor package, and regional epigenome correlation pattern are revealed and visualized by coMet Bioconductor package. Most of results are visualized by R. The whole analytic procedure can be used as guidance for handling multiple-omics datasets for future cohort genomic and epigenetic studies.

There are several confounding factors might influence including gender, ethnic and race etc. For example, gender variation has great impact on the risk of lung cancer [95, 96]. Specific gene mutation such as CYP1A1 significantly increased the risk of female lung cancer [97]. Among all American cigarette smokers, African and Native Hawaiians are the more susceptible to lung cancer than white, Asian and Latino Americans [98]. In addition, some patients had quitted smoking, or received targeted therapy in our datasets. These confounding factors should also be considered as well as smoking intensity. Important clinical information of the patients are summarized in Additional file 9: Table S3.

Dysregulation of gene CpG methylation is one of the hallmarks of human carcinogenesis, therefore making local and global epigenome serving as good sensor to response environmental impact, such as smoking patterns, duration and host immune response. In this study, 24 unique genes are identified their role as distinguisher between high and low intensive smoking habits and their CpG sites methylation shows strong correlation patterns. Based on all the results, it is possible to improve current LSCC early diagnosis and treatment by utilizing these genes.

## Supplementary information

**Additional file 1: Fig. S1.** Principle Components Analysis (PCA) of 299 cases of RNA-seq with LSSC patients clinical data. Green dots represent cases of low groups; Red dots represent cases of low groups. There is no significant difference between high and low smoking intensity in LSCC patients.

**Additional file 2: Fig. S2.** Principle Components Analysis (PCA) of 299 cases of methylation with LSSC patients clinical data. Blue dots represent cases of low groups; Red dots represent cases of low groups. There is no significant difference between high and low smoking intensity in LSCC patients.

**Additional file 3: Fig. S3.** Global DNA methylation status with Hierarchical clustering (a) DNA methylation hierarchical clustering (b) Beta value distribution of global DNA methylation.

**Additional file 4: Fig. S4.** Hyper/hypo methylated CpG islands status and transcriptional expression of 24 unique genes. (a) Represents DNA methylation status and gene ID in column plot (b) Represents DNA methylation status and gene ID in dot plot.

**Additional file 5: Table S1A.** The summary of GO terms for up-regulated genes by RNA-seq.

**Additional file 6: Table S1B.** The summary of KEGG pathways for up-regulated genes by RNA-seq.

**Additional file 7: Table S2A.** The summary of GO terms for down-regulated genes by RNA-seq.

**Additional file 8: Table S2B.** The summary of KEGG pathways for down -regulated genes by RNA-seq.

**Additional file 9: Table S3.** Summary of clinical information of 299 LSCC cases involved in this study.

## Data Availability

The datasets analyzed during the current study are available in the TCGA public data, https://www.cbioportal.org/ and https://portal.gdc.cancer.gov/ [21, 22].

## Abbreviations

AIRE: Autoimmune Regulator
CYP1A1: Cytochrome P450 Family 1 Subfamily A Member 1
DEGs: Differentially expressed genes
GO: Gene Ontology
GSTM1: Glutathione S-Transferase Mu 1
KEGG: Kyoto Encyclopedia of Genes and Genomes
LSCC: lung squamous cell carcinoma
NSCLC: non-small cell lung cancer
PENK: Proenkephalin
SLC6A3: Solute Carrier Family 6 Member 3
TCGA: The Cancer Genome Atlas
WWTR1/TAZ: WW Domain Containing Transcription Regulator 1
