# Supplementary figures and images for: Integrative analysis of genetic and epigenetic profiling of lung squamous cell carcinoma (LSCC) patients to identify smoking level relevant biomarkers
Source: BioData Min. 2019 Oct 21;12:18. doi: 10.1186/s13040-019-0207-y (PMC6802182; doi:10.1186/s13040-019-0207-y)

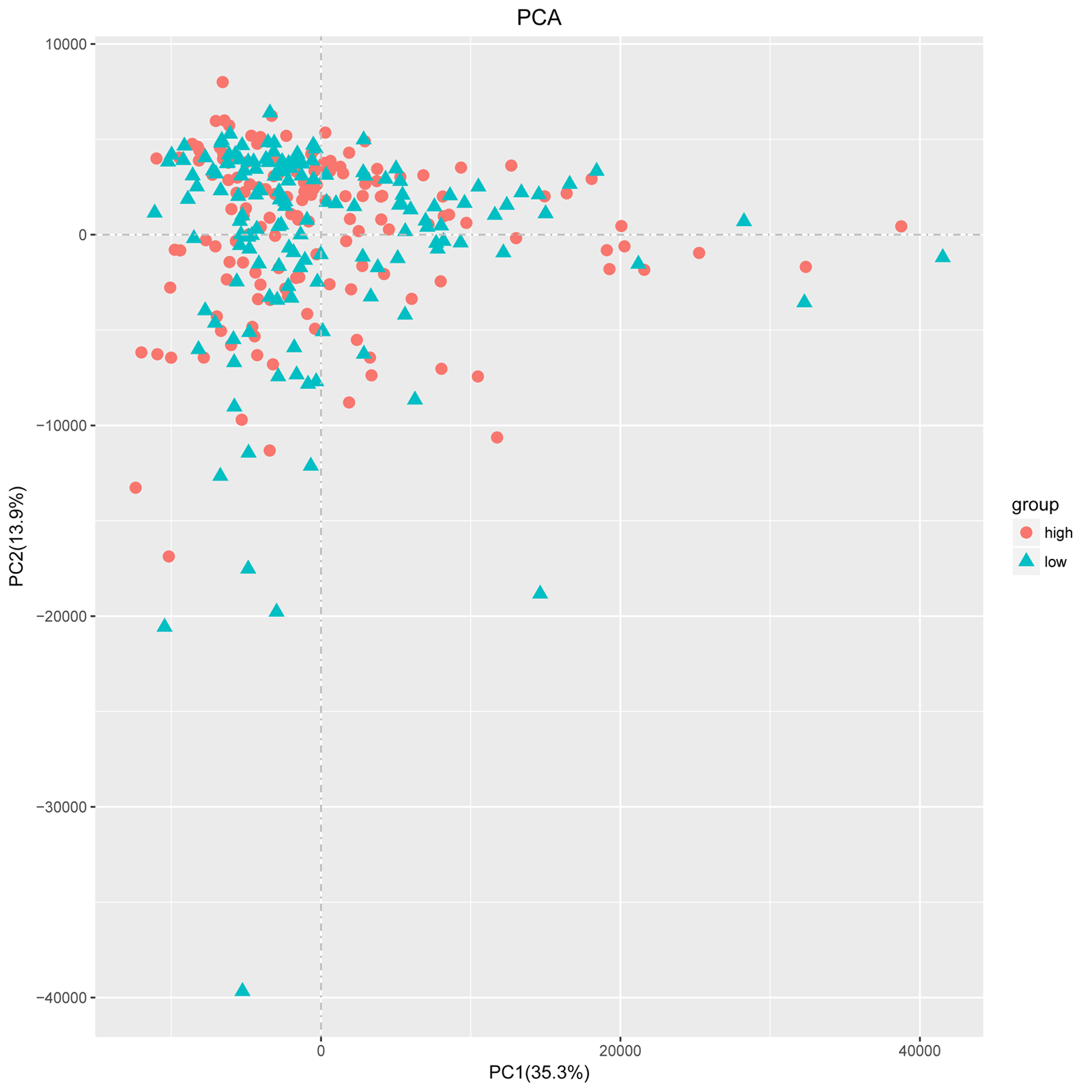

Supplement: Supplementary file 1 — Additional file 1: Fig. S1. Principle Components Analysis (PCA) of 299 cases of RNA-seq with LSSC patients clinical data. Green dots represent cases of low groups; Red dots represent cases of low groups. There is no significant difference between high and low smoking intensity in LSCC patients. [file 13040_2019_207_MOESM1_ESM.tif]

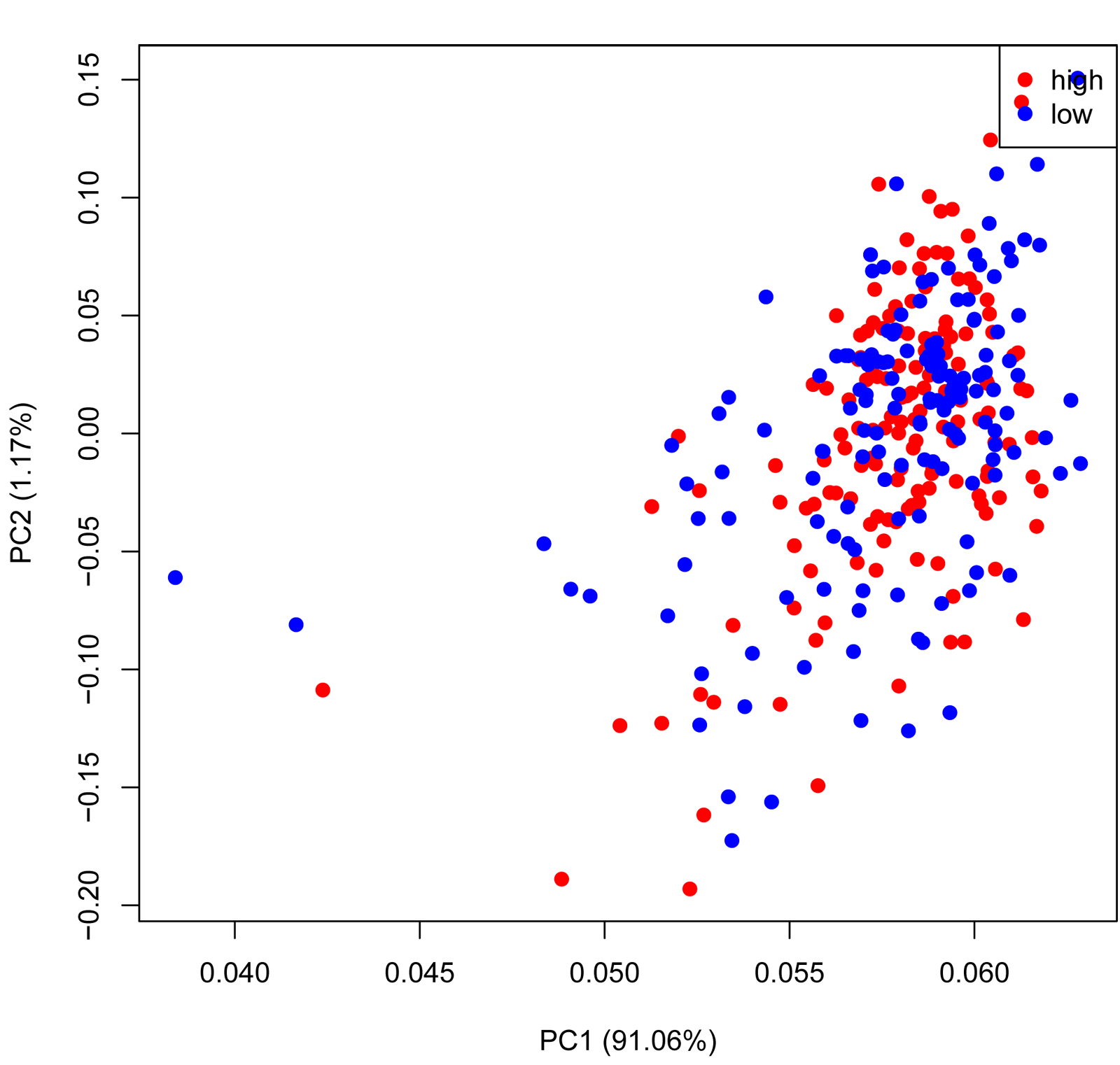

Supplement: Supplementary file 2 — Additional file 2: Fig. S2. Principle Components Analysis (PCA) of 299 cases of methylation with LSSC patients clinical data. Blue dots represent cases of low groups; Red dots represent cases of low groups. There is no significant difference between high and low smoking intensity in LSCC patients. [file 13040_2019_207_MOESM2_ESM.tif]

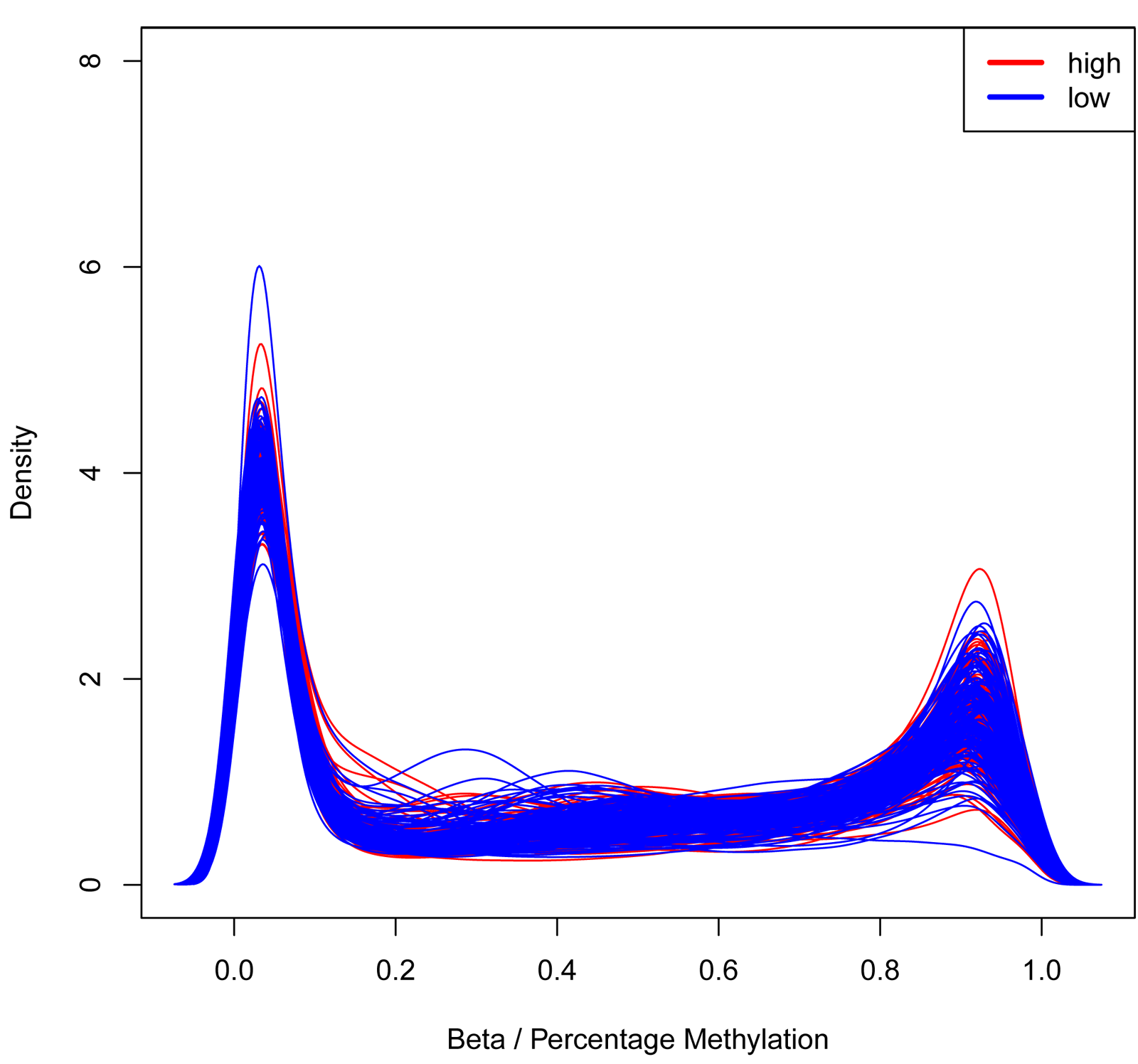

Supplement: Supplementary file 3 — Additional file 3: Fig. S3. Global DNA methylation status with Hierarchical clustering (a) DNA methylation hierarchical clustering (b) Beta value distribution of global DNA methylation. [file 13040_2019_207_MOESM3_ESM.zip › S3B_FigR1.tif]

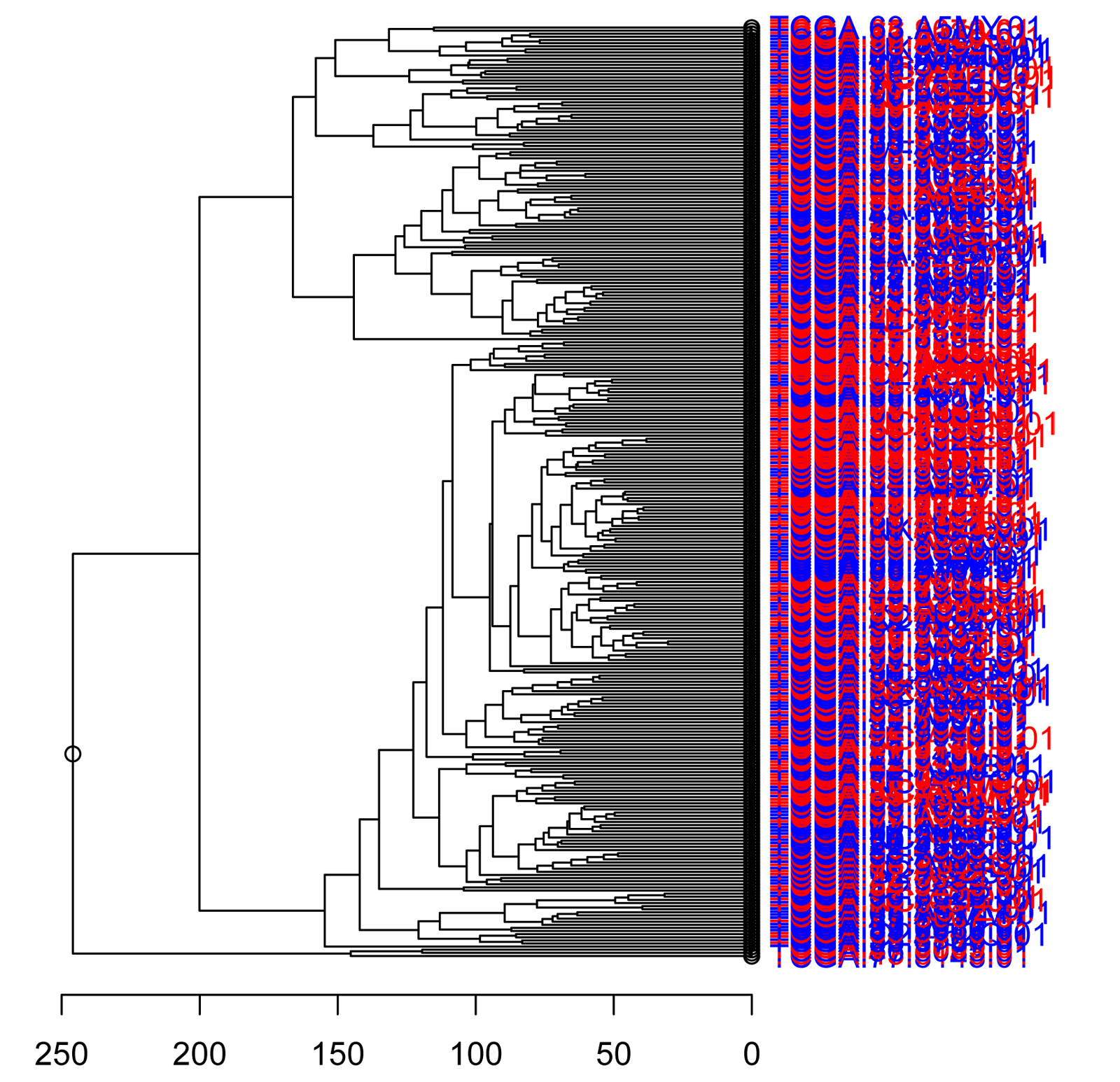

Supplement: Supplementary file 3 — Additional file 3: Fig. S3. Global DNA methylation status with Hierarchical clustering (a) DNA methylation hierarchical clustering (b) Beta value distribution of global DNA methylation. [file 13040_2019_207_MOESM3_ESM.zip › S3A_FigR1.tif]

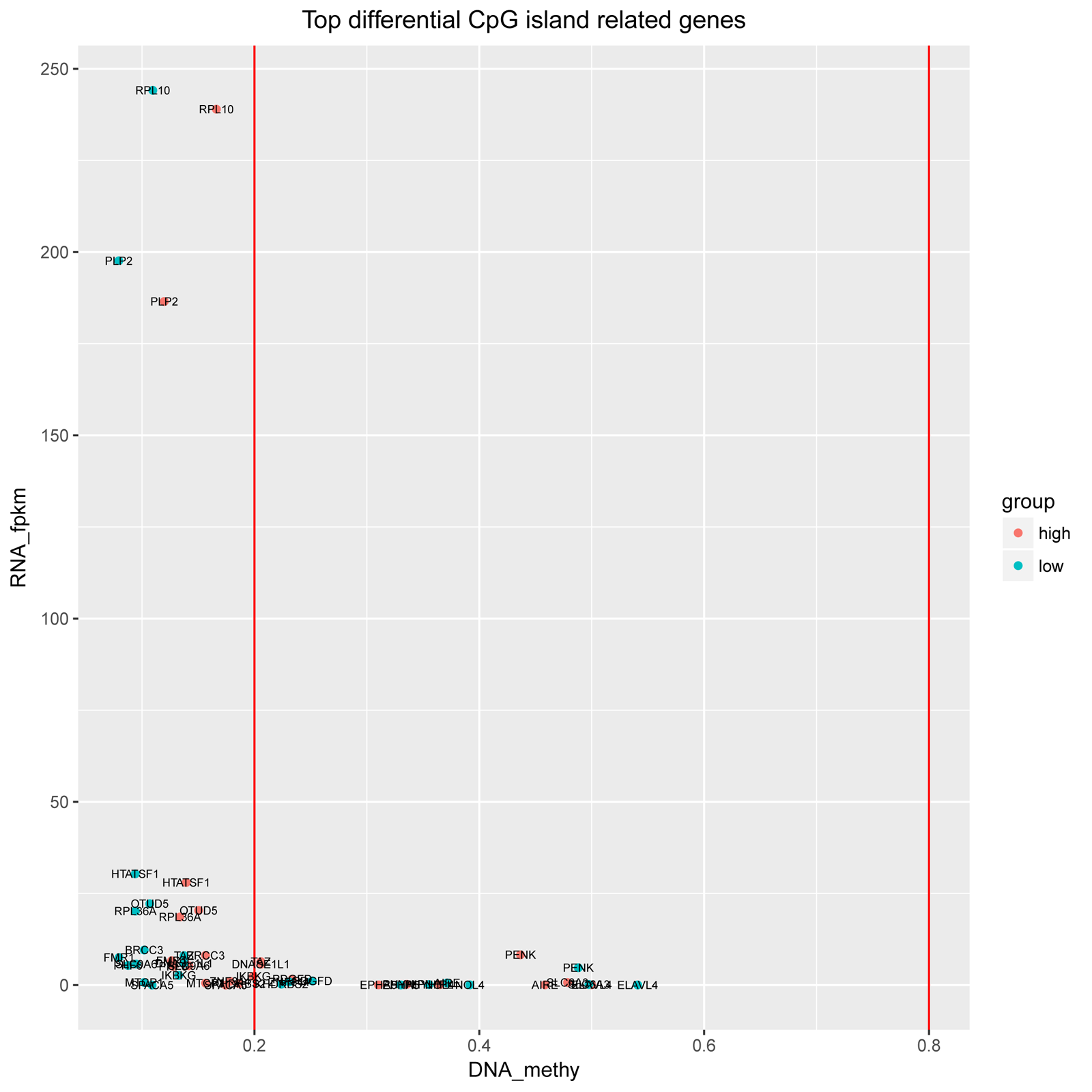

Supplement: Supplementary file 4 — Additional file 4: Fig. S4. Hyper/hypo methylated CpG islands status and transcriptional expression of 24 unique genes. (a) Represents DNA methylation status and gene ID in column plot (b) Represents DNA methylation status and gene ID in dot plot. [file 13040_2019_207_MOESM4_ESM.zip › S4B_FigR1.tif]

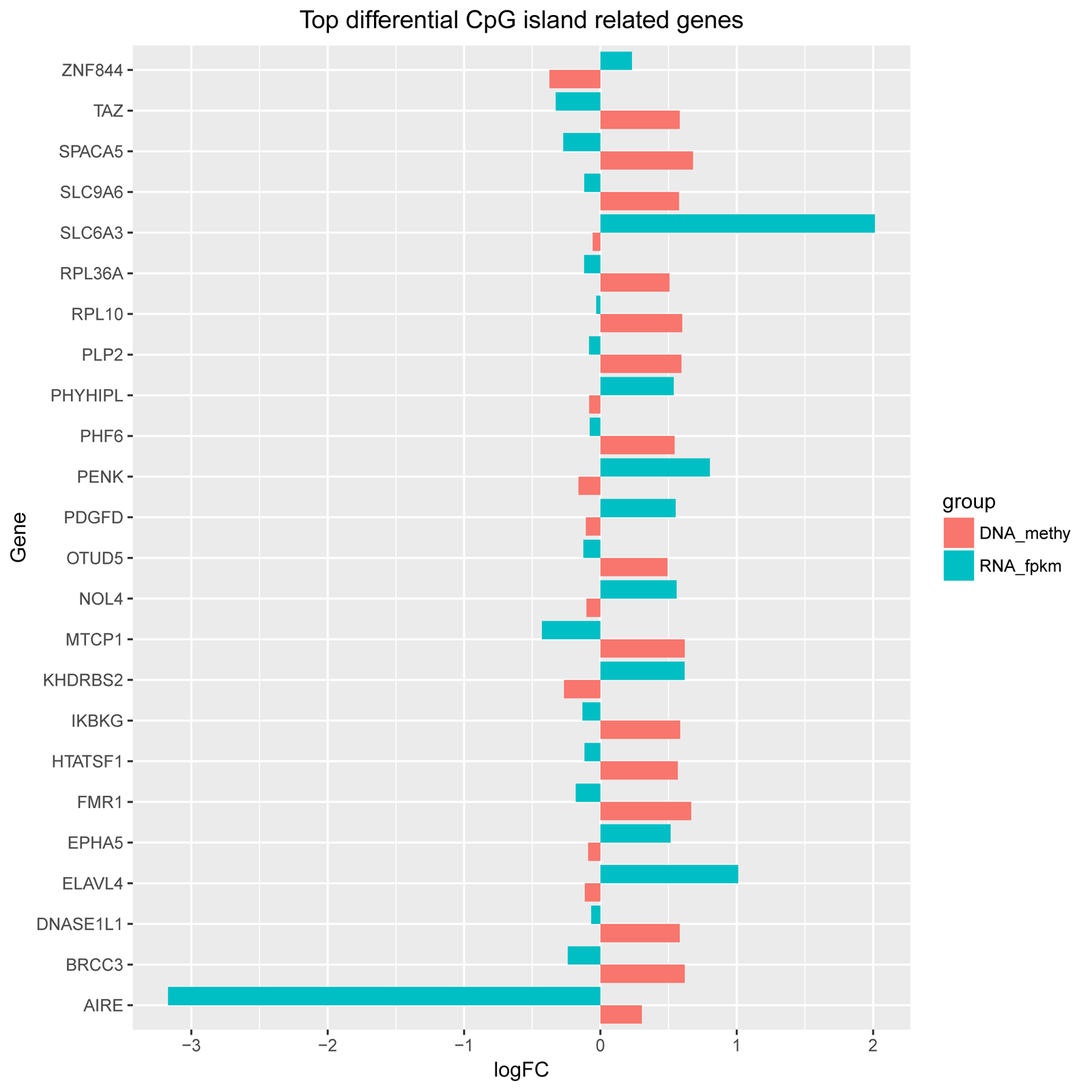

Supplement: Supplementary file 4 — Additional file 4: Fig. S4. Hyper/hypo methylated CpG islands status and transcriptional expression of 24 unique genes. (a) Represents DNA methylation status and gene ID in column plot (b) Represents DNA methylation status and gene ID in dot plot. [file 13040_2019_207_MOESM4_ESM.zip › S4A_FigR1.tif]
